# Human Leukocyte Antigen-DR Expression on Monocytes Is a Useful Predictor in a Systemic Inflammation Response-Based Prognostic Model in Advanced Non-Small Cell Lung Cancer

**DOI:** 10.3390/ijms26189226

**Published:** 2025-09-21

**Authors:** Gergő Szűcs, András Gézsi, Márton Szentkereszty, György Losonczy, Gábor Barna, Gabriella Gálffy, Anikó Bohács, Lilla Tamási, Veronika Müller, Edit I. Buzás, Zsolt I. Komlósi

**Affiliations:** 1 Department of Pulmonolgy, Semmelweis University, 1089 Budapest, Hungary; szucs.gergo@semmelweis.hu (G.S.); losonczy.gyorgy@semmelweis.hu (G.L.); bohacs.aniko@semmelweis.hu (A.B.); tamasi.lilla@semmelweis.hu (L.T.); muller.veronika@semmelweis.hu (V.M.); 2 Institute of Genetics, Cell- and Immunobiology, Semmelweis University, 1089 Budapest, Hungary; buzas.edit@semmelweis.hu; 3 Department of Artificial Intelligence and Systems Engineering, University of Technology and Economics, 1117 Budapest, Hungary; gezsi.andras@vik.bme.hu; 4 Department of Pathology and Experimental Cancer Research, Semmelweis University, 1085 Budapest, Hungary; szentkereszty.marton@semmelweis.hu (M.S.); barna.gabor@semmelweis.hu (G.B.); 5 Pulmonology Center of the Reformed Church in Hungary, 2045 Törökbálint, Hungary; ggalffy@hotmail.com; 6 HCEMM-SU Extracellular Vesicle Research Group, 1089 Budapest, Hungary; 7 HUN-REN SU Translational Extracellular Vesicle Research Group, 1089 Budapest, Hungary

**Keywords:** HLA-DR, systemic inflammation response index, non-small cell lung cancer, nomogram model

## Abstract

Inflammation and immune evasion promote tumorigenesis and progression. Elevated systemic inflammation response index (SIRI) is associated with poor progression-free survival (PFS) and overall survival (OS) in non-small cell lung cancer (NSCLC) patients. Low Human Leukocyte Antigen-DR (HLA-DR) expression on monocytes is also associated with poor prognosis in NSCLC. We aimed to investigate the relationship between these two indicators and develop a predictive model based on them. SIRI was calculated and monocyte HLA-DR expression was measured by flow cytometry in 58 advanced (stage IIIB-IV) NSCLC patients. The log-rank test and multivariate Cox proportional hazard regression model were used for analysis. We confirmed that both high SIRI and low monocyte HLA-DR expression were associated with poor PFS and OS, respectively. We found a significant inverse correlation between SIRI and monocyte HLA-DR expression. In the multivariable Cox regression model, both SIRI and monocyte HLA-DR expression were identified as independent prognostic markers for PFS and OS. We also developed a nomogram for predicting PFS and OS. In conclusion, we demonstrated that the systemic inflammation response of advanced NSCLC patients, estimated by SIRI, was associated with reduced HLA-DR expression on circulating monocytes, which may influence their antigen-presenting function. Consequently, the integration of these two biomarkers into one prognostic model improves short term survival prediction in advanced NSCLC. To our knowledge, this is the first integration of SIRI and HLA-DR into a combined prognostic nomogram.

## 1. Introduction

Non-small cell lung cancer (NSCLC) is a leading cause of cancer-related death worldwide [1,2]. It is partially explained by the fact that most of the patients with NSCLC are diagnosed at an advanced stage [3]. In Hungary, 52.4% of patients were diagnosed at stage IIIB-IV in 2024 [4]. Survival of advanced NSCLC patients is largely determined by their response to non-surgical oncotherapy: chemotherapy, radiotherapy, biological therapy (including immunotherapy), tyrosine kinase inhibitors, and various combinations thereof. Cytotoxic treatments reduce tumor mass, help to uncover hidden tumor (neo)antigens, and thus provide targets for the cellular immune responses [5,6,7]. The initiating event of an anti-tumor T cell response is the presentation of an antigen to T cells via major histocompatibility complex (MHC) molecules, in parallel with costimulatory or coinhibitory signals. Although the development of therapeutic antibodies blocking coinhibitory interactions (involving PD-1, PD-L1, or CTLA-4) has revolutionized anti-cancer immunotherapies [8], the role of MHC molecules themselves is much less well appreciated.

Tumor-induced chronic inflammation causes immune suppression and provides a favorable microenvironment for progression of malignant disease [9,10,11]. Several different indices are in use for assessing the immunological state of the patient and aiming to predict the prognosis of various malignancies [12]. Among these, the Lymphocyte-to-Monocyte Ratio (LMR) and systemic inflammation response index (SIRI) are the only two with any connection to antigen presentation, as their calculation takes into account the relative abundance of monocytes, the professional APCs in the peripheral blood. SIRI probably mirrors the imbalance of immune cells more reliably, as it has better predictive power [12], and it is one of the best biomarkers in advanced NSCLC [13]. However, not only the relative abundance but also the functional capability of APCs is critical in the initiation of an effective anti-tumor response [14,15,16].

We aimed to investigate the predictive utility of the expression of Human Leukocyte Antigen-DR (HLA-DR), the main class II MHC molecule on monocytes, in a systemic inflammation response-based prognostic model in advanced NSCLC. We succeeded in demonstrating for the first time that the systemic inflammation response of advanced NSCLC patients, estimated by SIRI, was associated with reduced HLA-DR expression on monocytes, an important element of immune disfunction.

## 2. Results

### 2.1. Patient Characteristics

**Figure 1:**
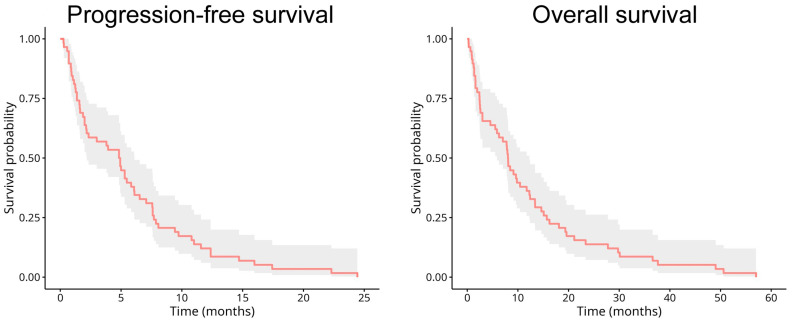
Kaplan–Meier estimates of progression-free survival (PFS) and overall survival (OS) of all patients. Gray background demonstrates 95% confidence intervals.

The study included 58 randomly selected patients with advanced-stage NSCLC (IIIB-IV) before starting oncological treatment. Demographic and clinical characteristics are shown in Table 1. Patient groups characterized by high and low levels of HLA-DR expression on monocytes were compared with each other. Although the sex distribution, age, smoking history, stage, and tumor histology were similar in the two groups, the body mass index was lower, the performance status was poorer, and SIRI was found to be higher in the low-HLA-DR-expression group. Patients in both groups received similar oncotherapy based on the decision of the same multidisciplinary team. The progression-free survival (PFS) and overall survival (OS) of all patients are shown in Figure 1.

### 2.2. Predictive Performance and Optimal Cut-Off Value Selection for the Biomarkers

**Figure 2:**
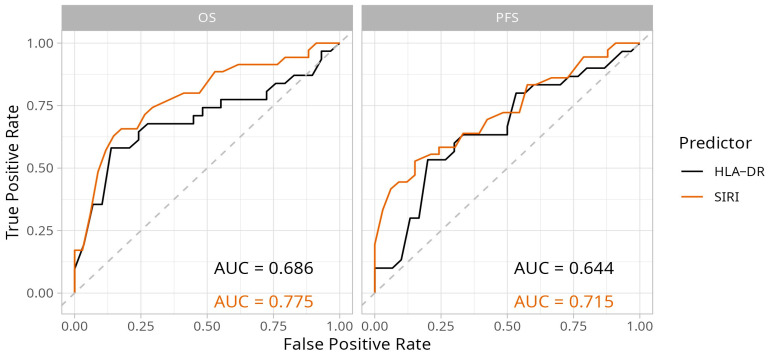
Receiver Operating Characteristic (ROC) curves showing the predictive performance of monocyte HLA-DR expression and systemic inflammation response index (SIRI) for progression-free survival (PFS) and overall survival (OS) of advanced NSCLC patients. HLA-DR expression of CD14+ monocytes was measured by using flow cytometry. Optimal cut-off values for monocyte HLA-DR expression and SIRI were determined based on these analyses. (AUC: area under the curve).

We used ROC curves to analyze the performance of HLA-DR expression on monocytes and SIRI as biomarkers in predicting longer-than-median PFS and OS (Figure 2). Optimal cut-off values were determined based on these analyses. Optimal cut-off values for this patient population were 36.53 for HLA-DR (median fluorescence intensity) and 4.0 for SIRI. Areas under the curves were well over 0.5 and were higher for prediction of OS (HLA-DR AUC: 0.686; SIRI AUC: 0.775) than PFS (HLA-DR AUC: 0.644; SIRI AUC: 0.715). SIRI was better than HLA-DR in predicting OS.

### 2.3. Relationship Between SIRI and Monocyte HLA-DR Expression

**Figure 3:**
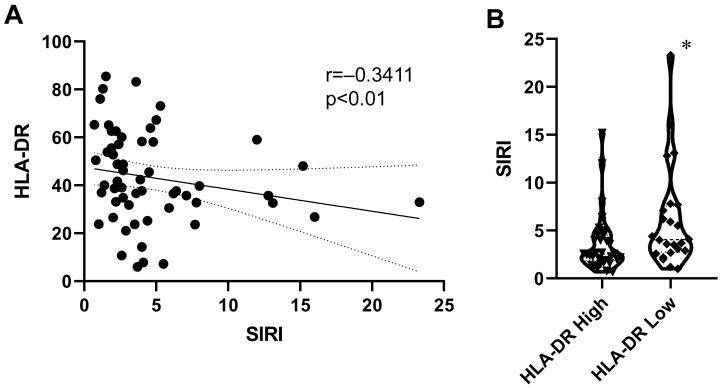
Interrelation of SIRI and monocyte HLA-DR expression. (**A**) Correlation between SIRI and monocyte HLA-DR expression (Spearman’s rank-order test). The trendline and its 95% confidence intervals (dotted lines) are for illustration. (**B**) Violin plot of SIRI values of patients with different monocyte HLA-DR expression levels (Mann–Whitney U test, *: *p* < 0.05). Patients were divided into HLA-DR High and HLA-DR Low groups using the optimal cut-off value (36.53), determined by analysis of the ROC curve (Figure 2, Youden index).

A weak but significant inverse correlation was found between SIRI and monocyte HLA-DR expression in advanced NSCLC patients (*p* < 0.01). Patients were divided into HLA-DR High and HLA-DR Low groups, using the optimal cut-off values of this biomarker. HLA-DR Low patients had significantly higher SIRI values (*p* < 0.05, Figure 3).

### 2.4. Prognostic Analysis

**Figure 4:**
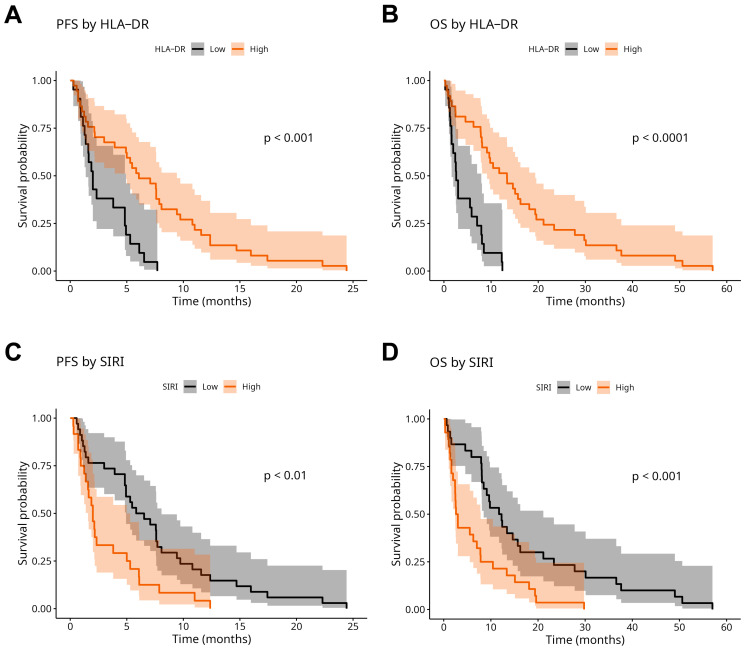
Kaplan–Meier estimates of progression-free survival (PFS) according to monocyte HLA-DR expression (**A**) and systemic inflammation response index (SIRI) (**C**). Kaplan–Meier estimates of overall survival (OS) according to monocyte HLA-DR expression (**B**) and SIRI (**D**). Curves were compared with the log-rank test. Gray and orange backgrounds demonstrate 95% confidence intervals.

Kaplan–Meier estimates were used for the analyses of PFS and OS according to monocyte HLA-DR expression and SIRI. Patients were divided into Low and High HLA-DR-expressing subgroups, as well as into Low and High SIRI subgroups, by using the optimal cut-off values (see above). The log-rank comparison of curves revealed significant differences in the survival and disease progression of these subgroups. We found that low HLA-DR expression and high SIRI values were associated with poor PFS and OS rates, respectively (Figure 4). Monocyte HLA-DR expression and SIRI were confirmed to be significant predictors in univariate Cox proportional hazard (PH) regression analysis, as well (Table 2). Moreover, in the multivariate Cox PH model, both of these biomarkers were identified as independent prognostic factors for PFS and OS (Table 2).

### 2.5. Nomograms for Predicting Progression-Free Survival and Overall Survival Rates in Advanced NSCLC

**Figure 5:**
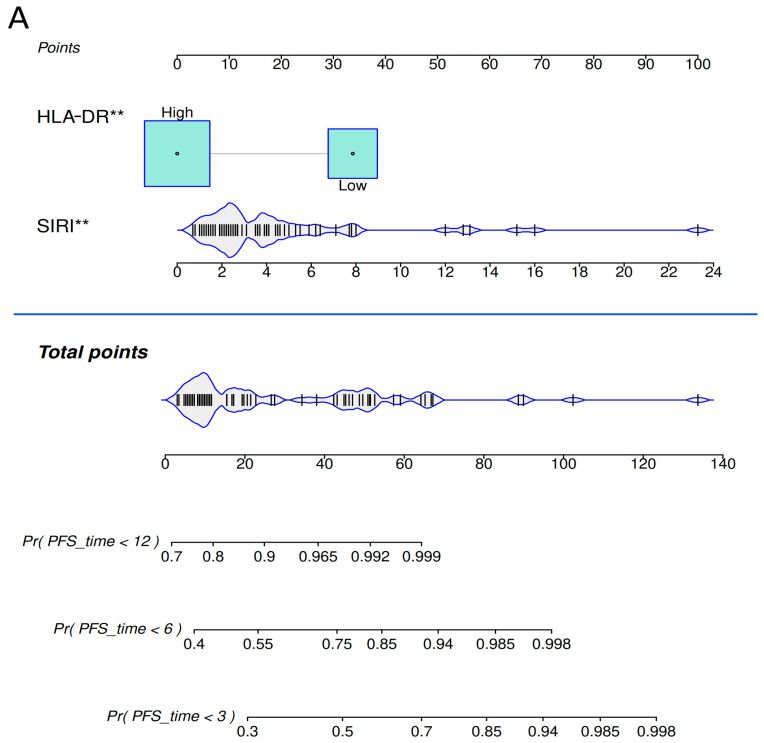

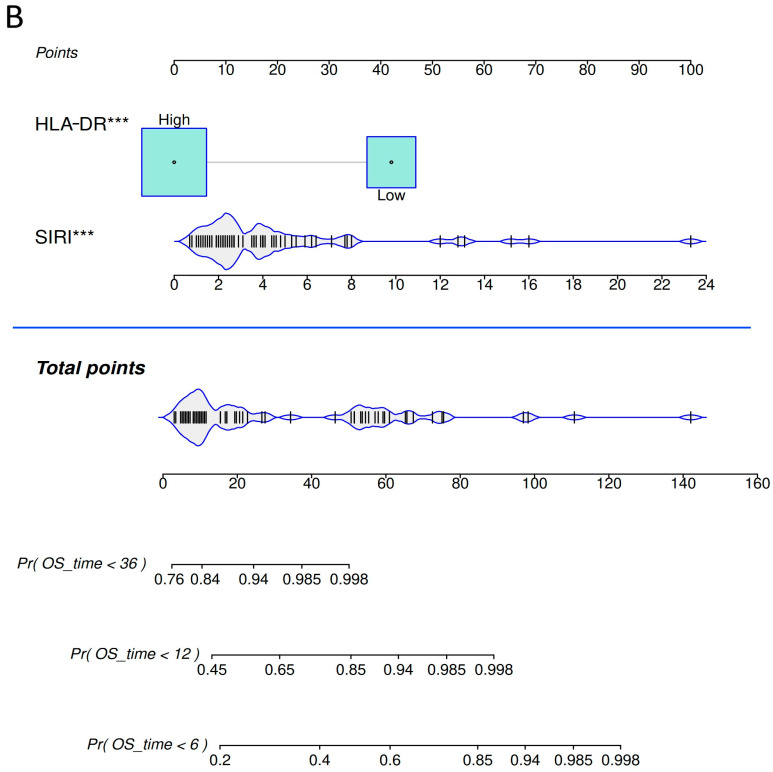
Nomograms for predicting PFS and OS of advanced NSCLC patients, based on multivariate Cox proportional hazard regression models. (**A**) Nomogram for prediction of 3-, 6- and 12-month PFS. (**B**) Nomogram for prediction of 6-, 12- and 36-month OS (Pr values denote the estimated probability that the PFS or OS time is less than the specified number of months; ** *p* < 0.01, *** *p* < 0.001).

We also developed nomograms based on the multivariate Cox PH model to facilitate individualized prediction of PFS and OS (Figure 5). First, patients can be categorized as Low- or High- HLA-DR-expressing by using the optimal cut-off value determined previously. Then, the HLA-DR High patients’ points can be read from the upper scale (HLA-DR Low patients receive zero points). In the next step, we calculate SIRI and read the points corresponding with the patients’ SIRI value also from the upper scale. Finally, we sum up the points of the patient and find it on the Total points scale (under the blue line). Via projection of a given total point to the downstream scales, patients’ individual probabilities (Pr) of reaching 3-, 6- and 12-month PFS (Figure 5A), and 6-, 12- and 36-month OS (Figure 5B) can be determined, respectively.

### 2.6. Model Performance and Validation

Post hoc power calculations indicated moderate to high power for the covariates included in the multivariable Cox models: for HLA-DR, the estimated power was 63.4% for progression-free survival and 88.8% for overall survival, while for SIRI, the corresponding values were 76.1% (PFS) and 81.6% (OS).

**Figure 6:**
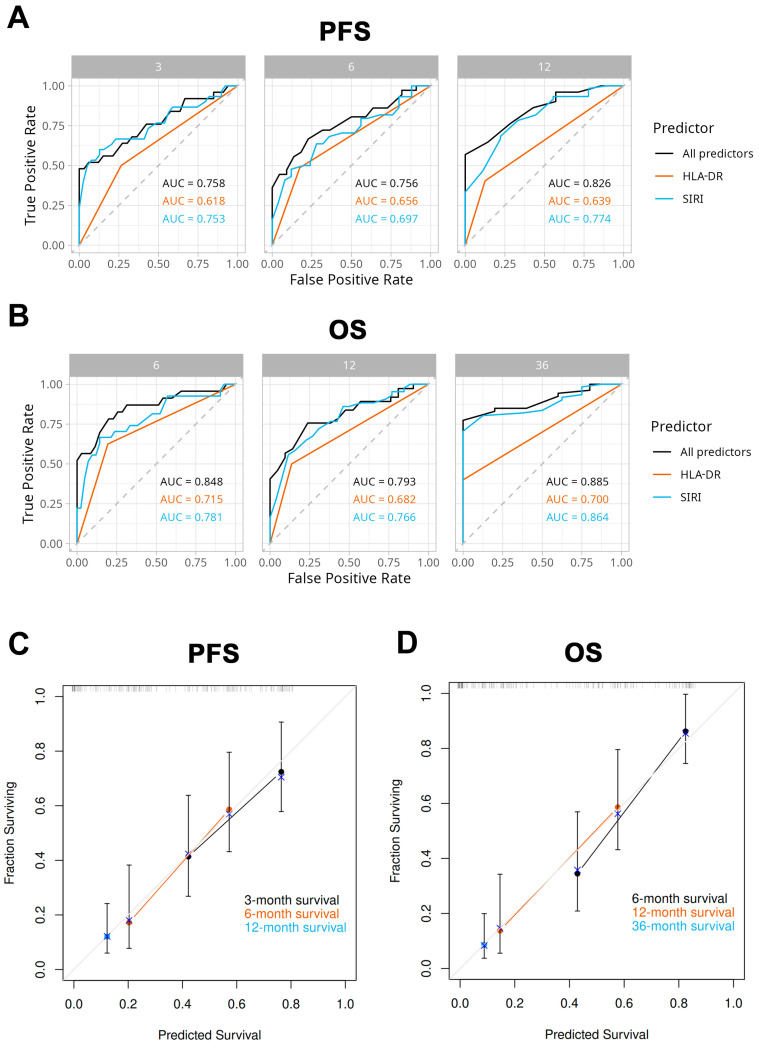
(**A**,**B**) ROC curves were plotted to evaluate the performance of our models in predicting 3-, 6- and 12-month PFS rate (**A**) and 6-, 12-, and 36-month OS rate (**B**) compared to monocyte HLA-DR expression and SIRI, respectively. All predictors: total points calculated by using PFS and OS nomograms (Figure 5), respectively (AUC: area under the curve). (**C**,**D**) Calibration curves. Predicted versus observed 3-, 6- and 12-month PFS rates (**C**) and 6-, 12-, and 36-month OS rates (**D**) were plotted to assess the goodness of fit of our model.

To assess the accuracy and additional benefit of our models, we compared ROC curves of the two predictors (HLA-DR expression and SIRI) per se, and the model total points (all predictors) for the 3-, 6-, and 12-month PFS rate (Figure 6A) and 6-, 12-, and 36-month OS rate (Figure 6B), respectively. The areas under the ROC curves (AUCs) for nomogram total points (shown in the Figure) were greater than 0.75 in all conditions and slightly bigger than each of the individual predictors alone. The OS model appeared to show the greatest advantage in short-term (6-month) survival prediction.

Internal validations of the models were performed by using a bootstrap resampling method (B = 1000). Predicted and observed 3-, 6- and 12-month PFS rates (C), as well as 6-, 12-, and 36-month OS rates were plotted against each other in calibration plots (Figure 6C,D). This way, we demonstrated a good agreement between model-predicted and actual OS and PFS rates.

## 3. Discussion

Low expression of HLA-DR on monocytes, sometimes called immune paralysis or immune depression, is an important indicator of immune disfunction that may also contribute to reduced immune responsiveness. It is a robust [17,18,19] and valuable prognostic marker in critically ill patients [20,21,22] associated with poor outcome, as well as in low-birth-weight newborns, associated with higher incidence and more severe infections [22,23]. It has been demonstrated that low monocyte HLA-DR expression also predicts poor prognosis in NSCLC [24]. The systemic inflammation reaction to the propagation of a malignant tumor is known to be ineffective in tumor elimination and rather promotes further tumor growth and dissemination, especially in advanced stages of the disease. Although a large part of the anti-tumor T cell response is mediated by CD8+ cytotoxic T cells, the role of CD4+ T helper cells is also well demonstrated [25]. Moreover, the fact that high monocyte HLA-DR expression is a good indicator of efficacy of immune checkpoint inhibitors underscore the importance of T helper cell-mediated responses [26]. Therefore, several easy-to-use indices are established to assess the immune status of the patients, and nomogram models have been developed for prediction of PFS and OS based on this [12,13,27,28,29]. However, to our knowledge, our current models are the first in which SIRI and monocyte HLA-DR expression have been integrated successfully into prognostic nomograms.

We confirmed in our patient cohort that high SIRI alone and low monocyte HLA-DR alone were associated with poor PFS and OS, respectively. Indeed, we have also found lower BMI and poorer performance status in HLA-DR Low patients compared to HLA-DR High patients. Moreover, a significant inverse correlation was revealed between SIRI and monocyte HLA-DR expression, suggesting that the loss of HLA-DR from the surface of monocytes is also a phenomenon in cancer-related systemic inflammation. Diminished HLA-DR expression is probably also observable on tissue macrophages [30,31,32]. In the multivariable Cox PH regression model, both SIRI and monocyte HLA-DR expression were identified as independent prognostic markers for PFS and OS. We also developed nomograms based on these models that may be useful for prediction of prognosis in NSCLC. Areas under the ROC curves of nomogram “total points” suggest the utility of our models. The main benefit of our OS model could be improved prediction of short-term survival (<6 month).

Consequently, our results suggest that the negative prognostic effect of tumor-induced systemic inflammation is manifested both in an imbalance of immune cells (estimated by SIRI) and the functional disability of antigen-presenting cells (estimated by monocyte HLA-DR expression). Considering the crucial role of antigen presentation function in the efficacy of therapeutic immune checkpoint inhibition, the inclusion of a functional marker (HLA-DR) in a fused indicator could be useful in complementing existing biomarkers, such as PD-L1 expression and tumor mutational burden, in guiding immunotherapy.

In clinical oncology, it is difficult to predict quick deterioration of the patients’ clinical state. At the same time, the most frequently asked question by patients with advanced disease is focused on their life expectancy. We learned from studies in patients receiving intensive care because of sepsis [32] and in premature newborns [23] that monocyte HLA-DR expression, as a biomarker, can predict short-term prognosis with relatively good accuracy [33].

Our model could be developed further via standardization of HLA-DR measurement on monocytes. Limitations of our research include the modest sample size and the lack of external validation. Further validation of our results in a larger cohort of NSCLC patients is needed to elucidate the utility of this model in predicting the efficacy of immune checkpoint inhibitors and short-term prognosis.

## 4. Materials and Methods

**Patients**

Fifty-eight NSCLC patients, diagnosed at the Department of Pulmonology, Semmelweis University, were included in the study. Consecutive patients with a confirmed NSCLC diagnosis and advanced stage (IIIB, IIIC, or IV) were enrolled just before initiation of pre-medication and anti-cancer therapy. None of the patients had received any glucocorticoids or antibiotics in the previous two months. Patients with other malignancies, autoimmune diseases, or immune deficiencies were excluded. Progression-free survival (PFS) was calculated as the time from the start of first-line treatment to disease progression or death. Progression was monitored every 3 weeks by clinical examination and chest X-ray, and if any signs or symptoms suggested progression, CT, PET-CT, and/or MRI were performed. Chest and upper abdominal CT scans were performed at least every 3 months. Overall survival (OS) was the time from the start of first-line treatment to death. Peripheral venous blood was obtained, and routine hematology was performed by a Sysmex XN-1000 analyzer (Sysmex Co. Kobe, Japan). Systemic inflammation response index (SIRI) was calculated as

$$SIRI=\frac{Neutrophil\;count\;(G/L)\times Monocyte\;count\;(G/L)}{Lymphocyte\;count\;(G/L)}$$

**Flow cytometry**

Peripheral blood samples were collected in tubes containing sodium-heparin (Vacuette, Greiner Bio-One, Frickenhausen, Germany). Peripheral blood mononuclear cells (PBMCs) were isolated by density gradient centrifugation using Ficoll (Biochrom, Berlin, Germany) within 3 h of sample collection. Samples were labeled, right after PBMC isolation, using the following anti-human antibodies: phycoerythrin (PE)-/Dazzle 594-conjugated anti-HLA-DR (L234) and PE-indotricarbocyanine (Cy7)-conjugated anti-CD14 (M5E2; both from BioLegend, San Diego, CA, USA). Flow cytometry data were acquired using a Navios instrument (Beckman Coulter, Brea, CA, USA) and were analyzed in Kaluza 2.3 software (Beckman Coulter). Median fluorescence intensity (MFI) values were used to estimate HLA-DR expression on monocytes. The flow cytometry gating strategy is shown in Supplementary Figure S1. Results of typical HLA-DR High and HLA-DR Low patients are demonstrated in the overlay histogram.

MFI values depend on the flow cytometer and its setup and the antibody clone and the fluorescent dye it is conjugated with. A cut-off value has to be established by each laboratory aiming to introduce this test into routine clinical practice. In our case, the cut-off value for monocyte HLA-DR MFI was about 74% of the HLA-DR MFI value of healthy volunteers (Supplementary Figure S2). This can be used as a guideline for establishing the test in any laboratory. Later, after measuring several patients, the cut-off value can be fine-tuned.

**Statistical analysis**

Data were analyzed with GraphPad Prism 8.0.1 (GraphPad Software, La Jolla, San Diego, CA, USA) software and R (version 4.4.1 [34]). The normality of the data distributions was examined by the Kolmogorov–Smirnov test. The Mann–Whitney U test was used for comparison between groups, and Spearman’s rank-order correlation was used for testing the relationship between variables. The categorical data were compared by chi-squared or Fisher’s exact tests. Associations between biomarkers and survival (PFS, OS) were tested in both univariate and multivariate models. Log-rank tests and univariate and multivariate Cox proportional hazard (PH) regression models were used for assessing the predictive power of variables. We developed a nomogram using the regplot package [35] for predicting PFS and OS, based on the multivariate Cox PH model. Internal validation of the multivariate Cox PH model was performed by using the bootstrap method with 1000 resamples and depicted via calibration curves showing predicted versus observed PFS and OS values. Survival diagrams were analyzed by the Kaplan–Meier estimator. Receiver Operating Characteristic (ROC) curves were created to assess the predictive performance of variables, and optimal cut-off values were determined by using the Youden index. In order to evaluate the adequacy of our sample size for detecting covariate effects, we performed post hoc power calculations for each variable included in the multivariable Cox proportional hazards regression models. Power was estimated using the powerSurvEpi package in R [36]. For each covariate, we specified the observed hazard ratio, while all other quantities were derived from the study dataset. The overall event probability was estimated at the median survival time, which we used as the time horizon for the power calculations. Calculations assumed a two-sided significance level of 0.05. The resulting estimates provide the probability of detecting the observed effect sizes under the proportional hazards assumption.

## 5. Conclusions

In conclusion, we demonstrated for the first time that the systemic inflammation response of advanced NSCLC patients, estimated by SIRI, is associated with reduced expression of HLA-DR on the surface of circulating monocytes. We also developed nomograms based on SIRI and monocyte HLA-DR expression to estimate their utility for predicting OS and PFS in such patients. Integration of HLA-DR has improved the prognostic efficacy of the model in predicting short-term survival. External validation of these results on larger, independent cohorts is required for clinical translation.

## Figures and Tables

**Table 1:** Demographic and clinicopathological characteristics of all non-small cell lung cancer (NSCLC) patients, and patients divided into HLA-DR High and HLA-DR Low groups using the optimal cut-off value (36.53) determined by analysis of ROC curves (see below in Figure 2).

Group	All Patients	HLA-DR Low	HLA-DR High	*p* Value
	n = 58	n = 22	n = 36	
Sex				0.999
male, n (%)	37 (63.8)	14 (63.6)	23 (64)	
female, n (%)	21 (36.2)	8 (36.4)	13 (36)	
Age (years)	66.08 ± 7.5	66.5 ± 7.2	66.1 ± 7.6	0.642
Smoking status, pack-year (IQR)	40 (30, 45)	40 (30, 44)	41 (37.5, 46.3)	0.311
Oncology stage, n (%)				0.266
IIIB + IIIC	22 (37.9)	6 (27.3)	16 (44.4)	
IV	36 (62.1)	16 (72.7)	20 (55.6)	
Tumor histotype, n (%)				0.774
adenocarcinoma	39 (67.2)	14 (63.6)	25 (69.4)	
squamous cell carcinoma	19 (32.8)	8 (36.4)	11 (30.6)	
ECOG state, n (%)				**<0.01**
0	35 (60.3)	10 (45.5)	25 (69.3)	
1	15 (25.9)	5 (22.7)	10 (28)	
2	7 (12.1)	6 (27.3)	1 (2.7)	
3	1 (1.70)	1 (4.5)	0 (0)	
Oncology therapy				0.386
chemotherapy	50 (86.2)	18 (81.8)	32 (88.8)	
radiotherapy	20 (34.5)	7 (31.8)	13 (36.1)	
biological therapy	24 (41.4)	12 (54.5)	12 (33.3)	
tyrosine-kinase inhibitor	4 (6.9)	0 (0)	4 (11.1)	
best supportive care	6 (10.3)	2 (9.1)	4 (11.1)	
BMI (kg/m^2^)	26.5 ± 4.8	23.78 ± 4.2	27.07 ± 4.9	**<0.05**
WBC (G/L)	12.17 ± 5	13.3 ± 5	11.34 ± 5	**<0.05**
Neu. count (G/L)	9.2 ± 4.6	10.4 ± 4.7	8.36 ± 4.4	**<0.05**
Ly. count (G/L)	1.87 ± 0.95	1.79 ± 1	1.9 ± 0.9	0.820
Mono. count (G/L)	0.75 ± 0.33	0.82 ± 0.4	0.68 ± 0.3	**<0.05**
Platelets (G/L)	356 ± 151	406 ± 187	320 ± 109	**<0.05**
SIRI	4.62 ± 4.2	6.4 ± 5.4	3.5 ± 3	**<0.05**
Mono. HLA-DR (MFI)	43.17 ± 18.8	25.38 ± 9.8	54.22 ± 13.8	**<0.001**
Median PFS month (IQR)	4.88 (1.58, 7.68)	2 (1.37, 5.2)	5.83 (1.6, 10.8)	**<0.01**
Median OS month (IQR)	8.05 (2.46, 15.8)	2.8 (1.6, 7.7)	11.73 (6.27, 21.13)	**<0.001**

BMI: (kg/m^2^): body mass index, WBC: white blood cell, Neu: neutrophil granulocyte, Ly: lymphocyte, Mono: monocyte, SIRI: systemic inflammation response index, Mono HLA-DR expression (MFI): monocyte HLA-DR expression (median fluorescence intensity), PFS: progression-free survival, OS: overall survival, IQR: interquartile range. The bold *p* values indicate significant associations.

**Table 2:** Univariate and multivariate Cox proportional hazard regression analyses of factors associated with progression-free survival (PFS) and overall survival (OS; n = 58).

Characteristics	SIRI		HLA-DR (Low vs. High)	
Univariate analysis (PFS)	Hazard ratio (95% CI)	*p* value	Hazard ratio (95% CI)	*p* value
	1.16 (1.08–1.25)	<0.001	3.12 (1.67–5.82)	<0.001
Multivariate analysis (PFS)	Hazard ratio (95% CI)	*p* value	Hazard ratio (95% CI)	*p* value
	1.13 (1.05–1.21)	0.002	2.56 (1.32–4.94)	0.005
Univariate analysis (OS)	Hazard ratio (95% CI)	*p* value	Hazard ratio (95% CI)	*p* value
	1.19 (1.10–1.27)	<0.001	4.56 (2.35–8.86)	<0.001
Multivariate analysis (OS)	Hazard ratio (95% CI)	*p* value	Hazard ratio (95% CI)	*p* value
	1.14 (1.06–1.23)	<0.001	3.66 (1.81–7.41)	<0.001

## Data Availability

The data that support the findings of this study are available upon request from the corresponding author, Zsolt I. Komlósi.

## Supplementary Materials

The following supporting information can be downloaded at: https://www.mdpi.com/article/10.3390/ijms26189226/s1.

## Abbreviations

The following abbreviations are used in this manuscript:

SIRI	Systemic Inflammation Response Index
PFS	progression-free survival
OS	overall survival
NSCLC	non-small cell lung cancer
HLA-DR	Human Leukocyte Antigen-DR
MHC	major histocompatibility complex
APCs	antigen-presenting cells
LMR	Lymphocyte-to-Monocyte Ratio
BMI	body mass index
WBC	white blood cells
IQR	interquartile range
ROC	Receiver Operating Characteristic
AUC	area under the curve
CT	computer tomography
MRI	magnetic resonance imaging
PET/CT	positron emission tomography
MFI	median fluorescence intensity
PH	proportional hazard
PBMC	peripheral blood mononuclear cells
