# Supplementary material for: Human Leukocyte Antigen-DR Expression on Monocytes Is a Useful Predictor in a Systemic Inflammation Response-Based Prognostic Model in Advanced Non-Small Cell Lung Cancer
Source: Int J Mol Sci. 2025 Sep 21;26(18):9226. doi: 10.3390/ijms26189226 (PMC12471102; doi:10.3390/ijms26189226)
Supplement: Supplementary file 1 [file ijms-26-09226-s001.zip › ijms-3830617-supplementary.pdf]

Supplementary figures

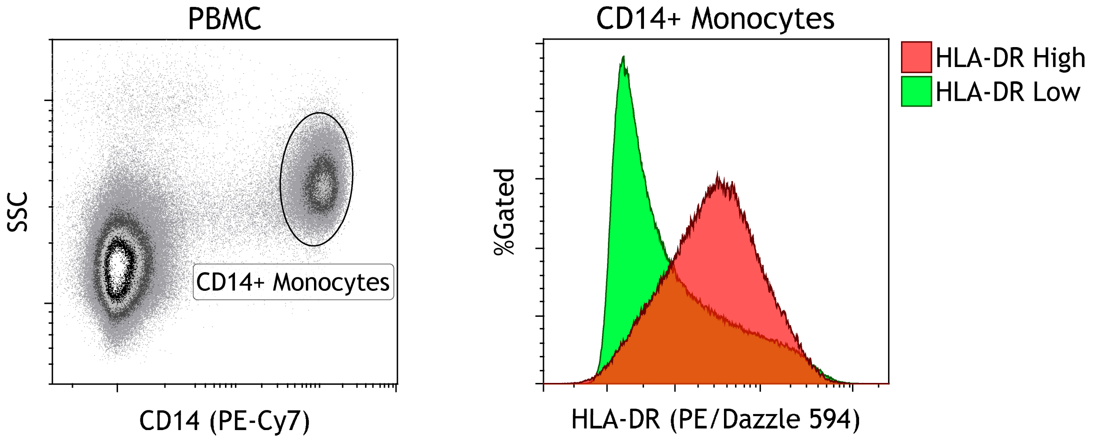

Supplementary Figure S1. Flow cytometry gating strategy (PBMC: Peripheral blood mononuclear cells).

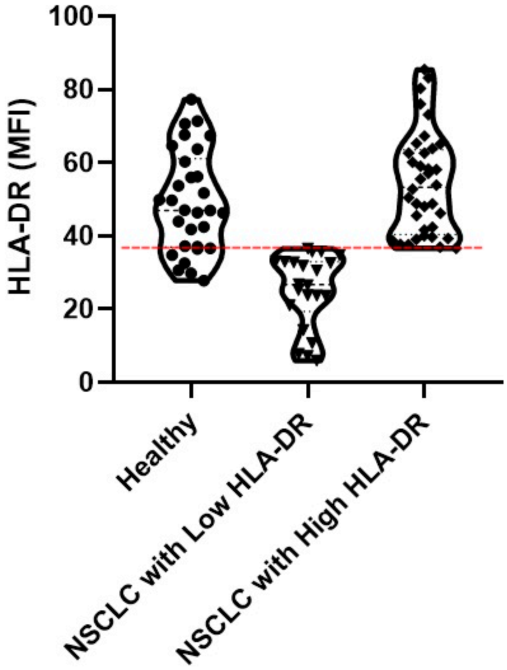

Supplementary Figure S2. Violin plot of monocyte HLA-DR expression levels of healthy and NSCLC patients. The red dashed line indicates the optimal cut-off values of monocyte HLA-DR expression (n=30 in Healthy, n=22 in HLA-DR Low, and n= 36 in HLA-DR High groups, one-way ANOVA test p<0.0001).
